# Supplementary material for: Identification of the Golden-2-like transcription factors gene family in Gossypium hirsutum
Source: PeerJ. 2021 Nov 16;9:e12484. doi: 10.7717/peerj.12484 (PMC8603818; doi:10.7717/peerj.12484)
Supplement: Supplemental Information 1 [file peerj-09-12484-s001.docx]

**Table S1:**

| Primer name | sequence (5' to 3') | Number of bases | Purification method |
| --- | --- | --- | --- |
| GLK1-F | CAATTACGTCGCCACCCTCT | 20 | PAGE |
| GLK1-R | CATCCGTTGATGGCGGTTTC | 20 | PAGE |
| GLK8-F | GGCTACCATTGCCATTAGGTC | 21 | PAGE |
| GLK8-R | AACGGCCGGAAGCATTGAT | 19 | PAGE |
| GLK38-F | CCGAACAGCAAACCCACATC | 20 | PAGE |
| GLK38-R | ACCATTGAAATTGGCGGTGG | 20 | PAGE |
| GLK39-F | GGAAATTGCTCGACGTTCACT | 21 | PAGE |
| GLK39-R | CTTGAGGCCTAAGGGCAAGG | 20 | PAGE |
| GLK46-F | TGCTTCTGCTGGTCTCGAAG | 20 | PAGE |
| GLK46-R | TGCAGGAAGTTCGGGTAAGG | 20 | PAGE |
| GLK47-F | CACACTTCAAACAGCAGGGC | 20 | PAGE |
| GLK47-R | TTCACATGTTGCTGCGCTTC | 20 | PAGE |
| GLK55-F | CATCCACCTTGCAACAACGG | 20 | PAGE |
| GLK55-R | ACCGGACTCATCATGGACAG | 20 | PAGE |
| GLK82-F | TTCGGCGGTTTCGAGTAGAC | 20 | PAGE |
| GLK82-R | CACGCGCTATGCACTTCATC | 20 | PAGE |
| GLK120-F | GGCAGCTCGATGCTTCCTCT | 20 | PAGE |
| GLK120-R | TTGGCTTTGCTGATGAAGGTG | 21 | PAGE |
| GhActin QF | ATCCTCCGTCTTGACCTTG | 19 | PAGE |
| GhActin QR | TGTCCGTCAGGCAACTCAT | 19 | PAGE |

**Use NCBI designed differentially expressed gene-specific primer sequences.**
